# Reply to Aljabali et al. Comment on “Abbas et al. The Safety and Efficacy of Nusinersen in the Treatment of Spinal Muscular Atrophy: A Systematic Review and Meta-Analysis of Randomized Controlled Trials. *Medicina* 2022, *58*, 213”

**DOI:** 10.3390/medicina58060793

**Published:** 2022-06-13

**Authors:** Basel Abdelazeem, Kirellos Said Abbas, James Robert Brašić

**Affiliations:** 1 Department of Medicine, McLaren Health Care, Flint, MI 48532, USA; 2 Department of Medicine, Michigan State University, East Lansing, MI 48532, USA; 3 Faculty of Medicine, Alexandria University, Alexandria 22621, Egypt; kirellossaid98@gmail.com; 4 Section of High Resolution Brain Positron Emission Tomography Imaging, Division of Nuclear Medicine and Molecular Imaging, The Russell H. Morgan Department of Radiology and Radiological Science, The Johns Hopkins University School of Medicine, Baltimore, MD 21287, USA; jbrasic1@jh.edu

We appreciate Ahmed Sami Aljabali and his colleagues for their interest and comments [1] regarding our article entitled “The Safety and Efficacy of Nusinersen in the Treatment of Spinal Muscular Atrophy: A Systematic Review and Meta-analysis of Randomized Controlled Trials” [2]. Here, we provide answers and further clarifications regarding their comments. 

First, regarding our finding on the motor improvement of patients with different types of SMA and nusinersen safety. We pooled the data from Acsadi et al. [3] that evaluated patients with infantile-onset (type 1 spinal muscular atrophy (SMA)) and later-onset SMA (type 3 and 4 SMA); from Finkel et al. [4] that evaluated SMA patients with type 1; and finally, from Mercuri et al. [5] that evaluated patient with SMA type 2 [5]. We are aware that all trials included different SMA types, and we mentioned it clearly in the Limitations section “Our meta-analysis was limited by the discrepancies in the study designs of the selected RCTs. Although the ENDEAR trial of only participants with infantile-onset SMA used both the HINE-2 score and the Children’s Hospital of Philadelphia Infant Test of Neuromuscular Disorders (CHOP INTEND) score to measure the motor milestone response, the EMBRACE trial of participants with both with both infantile-onset and later-onset SMA and the other phase 3 trial, the CHERISH trial, of only participants with later onset SMA used only the HINE-2 score”. However, that does not prevent us from performing meta-analyses on nusinersen and reporting the initial data on the overall efficacy and safety, especially since we discussed that points in detail in our discussion “To our knowledge, this is the first study to meta-analyze the safety and efficacy of nusinersen in SMA and to determine the significance of the results and the impact of the new data on clinical practice”, “Future investigations will be enhanced by the use of identical inclusion criteria and outcome measures. More stringent criteria for the selection of articles, including ages and rating scales, will enhance future reviews when there are many more articles to include”, and “A further limitation of the present meta-analysis is the small number of available RCTs with outcomes that were suitable for pooling through meta-analysis; thus, more RCTs are needed before making clinical recommendations based on these studies. Currently, one more RCT is in the recruiting stage and assessing the same outcomes (NCT04089566)”.

In regard to Figure 3, we did not mistakenly reverse the forest plot labels for the left and right directions, as mentioned by Aljabali et al. We clearly labeled our control group on the left side of the table within the forest plot, which corresponds to the left side of the figure; similarly, for the nusinersen group, we added our events and total data on the right side of the table within the forest plot, which corresponds to the right side of the figure. If we reversed the labels, then the control group would show positive outcomes, which are incorrect. Therefore, our current figure is correct and was not mistakenly reversed.

Second, Aljabali et al. claimed that throughout the whole article, we acknowledged that nusinersen is safe and effective for SMA treatment and discussed three points that were of their concern, but those points were extensively discussed and evaluated in the Discussion section in Section 4.2 adverse effects. We clearly mentioned in our conclusions that our initial results for the safety and efficacy of nusinersen are just promising, and we reported in multiple sections throughout our manuscript that more RCTs are still needed before making clinical recommendations based on included RCTs [3,4,5]. The three points of concern for Aljabali et al. and our replies are as follows:

(1) The efficacy of nusinersen in some SMA subgroups such as respiratory insufficiency, bulbar manifestations, gastric feeding tube, severe contractures or severe scoliosis, or medical disability. The exclusion criteria mentioned by Aljabali et al. were also mentioned in Mercuri et al. [5]. On the other hand, Finkel et al. [4] included patients with and without gastrostomy, as mentioned in the Supplementary Materials, and Acsadi et al. [3] reported exploratory efficacy endpoints that involved a change from baseline in ventilator use (including bilevel positive airway pressure, tracheostomy, and endotracheal tube), which means that he included patients with respiratory insufficiency. These examples go against Aljabali et al.’s comment that the clinical trial excluded those patients. Due to the lack of data and the few RCTs published so far, more data are still needed before delivering conclusion about those patients subgroup. We also mentioned that in the Limitations section “A further limitation of the present meta-analysis is the small number of available RCTs with outcomes that were suitable for pooling through meta-analysis; thus, more RCTs are needed before making clinical recommendations based on these studies”.
(2) Adult SMA patients. As indicated in our manuscript title, we only included infantile patients, and we did not comment on adult patients throughout our manuscript.
(3) The long-term efficacy and safety of nusinersen. We also reported that in the Limitations section, and we conducted our meta-analysis based on available RCTs.

Third, Aljabali et al. commented on abeparvovec-xioi (Zolgensma) and risdiplam (Evrysdi) as other disease-modifying therapies for SMA. We did not comment on those medications in our review. Future research is still needed to evaluate the efficacy and safety of those medications and to compare it with nusinersen. We recommend conducting a network meta-analysis or investigating the superiority or non-inferiority of the drugs mentioned with nusinersen to ensure cost-effectiveness. Erdos et al. [6], in their systematic review on the three abovementioned drugs, reported a lack of mid- and long-term data on risdiplam and improvement with nusinersen with all types, especially type 1.

Finally, Aljabali et al. agreed with us that nusinersen has a promising efficacy and safety data in children with SMA types 1, 2, and 3 who fall within the selection criteria of the included RCTs in our meta-analysis. Additionally, as mentioned in our manuscript and as Aljabali et al. mentioned in their comments, more RCTs with larger patient populations and longer follow-up durations are still needed before definitive recommendations can be made.

## Data Availability

All data included in this report are identified within the text, tables, and figures.
